# Atlas of *Schistosoma mansoni* long non-coding RNAs and their expression correlation to protein-coding genes

**DOI:** 10.1093/database/bay068

**Published:** 2018-07-09

**Authors:** Elton J R Vasconcelos, Vinícius C Mesel, Lucas F daSilva, David S Pires, Guilherme M Lavezzo, Adriana S A Pereira, Murilo S Amaral, Sergio Verjovski-Almeida

**Affiliations:** 1 Laboratório de Expressão Gênica em Eucariotos, Instituto Butantan, 05503-900 São Paulo, SP, Brazil; 2 Departamento de Bioquímica, Instituto de Química, Universidade de São Paulo, 05508-900 São Paulo, SP, Brazil; 3 College of Veterinary Medicine, Western University of Health Sciences, Pomona, CA 91766-1854, USA

## Abstract

Long non-coding RNAs (lncRNAs) have been widely discovered in several organisms with the help of high-throughput RNA sequencing. LncRNAs are over 200 nt-long transcripts that do not have protein-coding (PC) potential, having been reported in model organisms to act mainly on the overall control of PC gene expression. Little is known about the functionality of lncRNAs in evolutionarily ancient non-model metazoan organisms, like *Schistosoma mansoni*, the parasite that causes schistosomiasis, one of the most prevalent infectious-parasitic diseases worldwide. In a recent transcriptomics effort, we identified thousands of *S. mansoni* lncRNAs predicted to be functional along the course of parasite development. Here, we present an online catalog of each of the *S. mansoni* lncRNAs whose expression is correlated to PC genes along the parasite life-cycle, which can be conveniently browsed and downloaded through a new web resource http://verjolab.usp.br. We also provide access now to navigation on the co-expression networks disclosed in our previous publication, where we correlated mRNAs and lncRNAs transcriptional patterns across five life-cycle stages/forms, pinpointing biological processes where lncRNAs might act upon.

Database URL: http://verjolab.usp.br

## Background and summary

RNA-Seq has been proven to be a valuable technique for gene expression studies in important neglected tropical disease-causing agents, such as *Schistosoma mansoni* (1–4). This is a flatworm parasite endemic in both Africa and South America continents that causes schistosomiasis, an infectious disease that affects, together with other schistosome species, over 250 million people worldwide (5). Due to its complex life cycle, this parasite is also an interesting model for investigations in the fields of genomics and transcriptomics [please refer to the following World Health Organization (WHO) website for a detailed view of schistosomes life cycle: http://www.who.int/schistosomiasis/epidemiology/en/]. High-throughput screenings using RNA-Seq have already shown that there are drastic changes in gene expression during parasite development, with thousands of differentially expressed genes identified in different life stages (4, 6–8).

Transcriptomics efforts in several eukaryotic organisms (including pathogens) have revealed a large amount of long non-coding RNAs (lncRNAs) in their genomes (7, 9–13). Looking briefly at the GENCODE current release statistics for the human genome (version 28— https://www.gencodegenes.org/stats/current.html), we see that the number of already mapped lncRNAs (15,779) plus small ncRNAs (7569) has already surpassed the number of protein-coding (PC) genes (19,901), indicating the importance of these molecules. Regarding their dynamics, several studies have unraveled lncRNAs as key regulators in multiple crucial pathways, mainly those involved in epigenetics mechanisms and control of gene expression at both transcriptional and post-transcriptional levels (14, 15) (and references therein). Nevertheless, the whole deciphering of their *in vivo* functionality is still on its infancy.

We recently reported the identification of novel 7029 canonically-spliced putative long intervening non-coding RNAs (lincRNAs) and 402 spliced lncRNAs that are antisense to PC genes within the *S. mansoni* genome (7). One can navigate on those novel genes loci and check their architectures through the *S. mansoni* UCSC-like genome browser (http://schistosoma.usp.br/), which we developed previously (6, 7). Several hundreds of *S. mansoni* lincRNAs (SmLINC) showed traits for being functional (7), such as the presence of trimethylation on lysine 4 of histone H3 (H3K4me3) as an epigenetic mark at their transcription start sites (2878/7029), evolutionary conservation among *Schistosoma haematobium* and *Schistosoma japonicum* (3453/7029), and differential expression across five different life-cycle stages of the parasite (916/7029).

Following a practice adopted by others on raising hypotheses about lincRNA functions (16–18), we also used the ‘guilt by association’ approach and built PC-lincRNA co-expression networks, on the attempt of unraveling key biological processes where lincRNAs might be involved during parasite development (see Figures 6 and 7; Supplementary Figures S3 and S5 from our previous work) (7). For generating those networks, we initially performed a global pair-wise expression correlation analysis (all genes against all) using normalized read-counting values (TPM) for each gene (PC and SmLINC) from the fifteen RNA-Seq libraries that were selected as described in our previous work (7) and represent the five life cycle stages: biological triplicates for cercariae (ERR022872, ERR022877 and ERR022878), somula 3 h (ERR022874, ERR022876 and ERR022879), somula 24 h (ERR022880, ERR022881 and ERR022882), male (SRR5170192, SRR5170191 and SRR5170190) and female (SRR5170180, SRR5170179 and SRR5170178) adults. The three former stages are from Protasio *et al.* (4), while adult worm samples are from our group (NCBI BioProject ID: PRJNA361136) (see Materials and methods herein and from our previous work) (7). The Pearson correlation data are the central core for all the newly-formatted atlas that we present in the current article. We now provide the parental source as a huge table of 4 columns by 31,073,159 rows, where each row corresponds to a significant (*P* < 0.05) pair-wise correlation (PC-PC, PC-SmLINC or SmLINC-SmLINC) (https://doi.org/10.6084/m9.figshare.5797257). The first two columns are the IDs from the pair of genes, whereas the third and fourth are the correlation score (*r*) and *P*-value, respectively.

**Figure 1.**
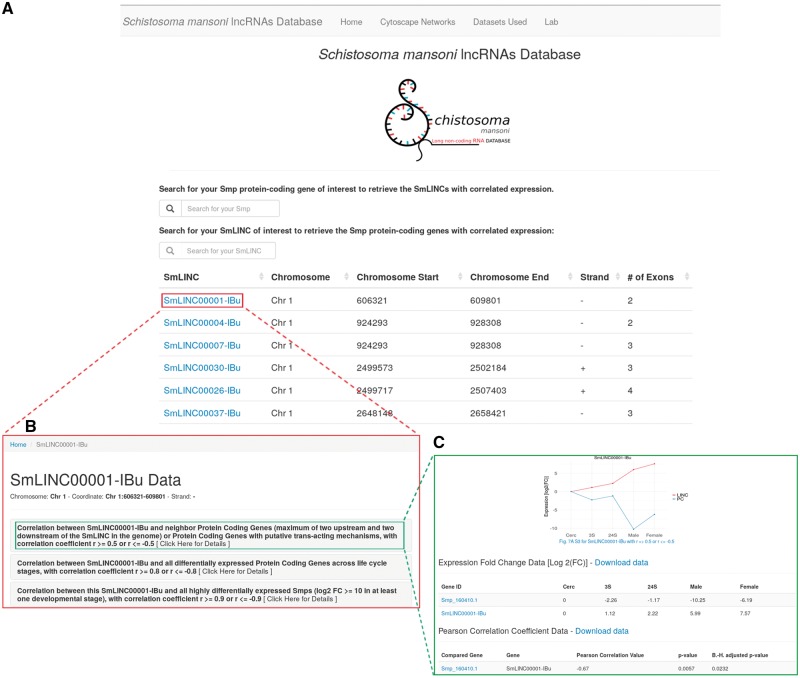
*Schistosoma mansoni* long non-coding RNAs database. The developed web resource disclosed herein allows the users to navigate on SmLINC RNA genes’ architecture and co-ordinates into the *S. mansoni* genome (**A**), as well as to pick the expression correlation coefficient values (*r*) for the pairs SmLINC/Smp (**B**) and visualize the SmLINC co-expression profile with protein-coding genes along five parasite’s developmental stages (**C**).

We call the data disclosed herein an atlas of *S. mansoni* lncRNAs, more precisely lincRNAs, antisense ncRNAs and their expression correlation coefficients (*r*) to PC genes along the parasite life-cycle. Through the newly developed web resource (http://verjolab.usp.br), the *S. mansoni* research community will be able to access the expression profiles of SmLINC genes of interest along with their either positively or negatively correlated Smp PC gene mates across the five parasites’ developmental stages (Figure 1).

We are also co-providing with this publication the full co-expression networks files in the Cytoscape (19) .cys format as figshare objects (https://doi.org/10.6084/m9.figshare.5797290), instead of static images that were disclosed in our previous work (7). The network .cys files can also be downloaded from the link ‘Cytoscape Networks > Other Cytoscape Networks’ at the uppermost bar in the website. The user is now able to navigate on those networks, visualizing the subclusters of correlated Smp-SmLINCs and then checking the co-expression profile of the target subclusters through the web resource released herein. For instance, once finding either a Smp gene or GO term of interest within the cytoscape co-expression network, one will identify the SmLINC(s) connected to that Smp or GO (i.e. those lincRNAs that are significantly co-expressed with the PC gene in question). Next, in the SmLINCs search tool from the web resource, the user can obtain and visualize further information about the identified SmLINC and its correlated Smp, such as their expression levels along five life-cycle stages of the parasite plus the expression Pearson correlation coefficient (*r*) with its statistical significance *P*-value, along with the Benjamini–Hochberg adjusted *P*-value. Additional Smps that are correlated to that SmLINC within the same subcluster are displayed. Also, any PC gene of interest can be directly searched at the Atlas of SmLINCs web resource by its Smp gene ID number; the correlated SmLINCs are displayed.

**Figure 2.**
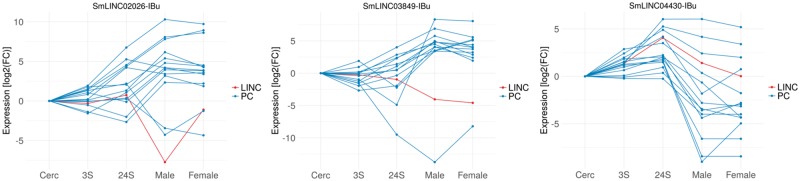
Co-expressed Smp-SmLINC subclusters along the five parasites’ developmental stages raise hypothesis on *S. mansoni* lncRNAs functionality. The three charts depict both positively and negatively correlated Smp-SmLINC pairs.

Three examples of Smp-SmLINC co-expression subclusters are depicted in Figure 2, where one may hypothesize about a SmLINC positive or negative regulatory activity on a set of PCs and, thus, experimentally test it in the lab. It is now known that inhibition of specific lncRNAs may modulate entire intracellular pathways. RNA interference (RNAi), antisense oligonucleotides (ASO) and ribozymes are examples of lncRNA-inhibitor compounds under current trial in human medicine (20).

In another example, the user can look up the genomic mapping of a PC gene of interest at the *S. mansoni* UCSC-like genome browser (http://schistosoma.usp.br/), which is searchable by the Smp gene ID number. Thus, the user will be able to inspect whether there is any SmLINC gene mapped to a genomic locus in the vicinity of the PC gene in question. With the neighbor SmLINC gene ID at hand, the user can retrieve it from the Atlas of SmLINCs web resource searching tool, if the SmLINC has a co-expression correlation with the neighbor PC gene of interest. For the correlation analyses, we have considered as neighbors the first two PC genes upstream and two downstream of each SmLINC gene in the genome. Any PC gene of interest can also be directly searched at the Atlas of SmLINCs web resource by its Smp gene accession number. The expression levels of the neighbor PC gene-SmLINC pair along the five life-cycle stages, the expression correlation coefficient, the correlation *P*-value and the Benjamini–Hochberg adjusted *P*-value (B.-H. adjusted *P*-value) can be visualized and/or downloaded as a chart and a table at the SmLINCs web resource. Evidence of co-expression correlation of a neighbor PC gene and a SmLINC pair along five different life-cycle stages of the parasite raises an experimentally testable hypothesis that the SmLINC RNA might modulate the expression of its neighbor Smp PC gene.

The user can also view and navigate through a network of the enriched biological processes, molecular functions and cellular components GO terms for PC genes co-expressed with lincRNAs, where lincRNAs might act upon. Our data represent the first reported set of structurally annotated *S. mansoni* lncRNAs. The data raise hypotheses about lincRNAs functionality through co-expression networks construction and assessment.

## Materials and methods

Detailed methods for (i) *S. mansoni* lncRNAs identification, (ii) differential expression and correlation analyses across the five developmental stages and (iii) co-expression network construction and analyses can all be found under the ‘Electronic supplementary material - Supplementary Methods’ section from our related work (7). More specifically, the Pearson correlation coefficient (*r*) with its statistical significance test *P*-value were calculated with the cor.test function within the R environment (version 3.3.2) as indicated in our previous work (7). The Benjamini–Hochberg adjusted *P*-value (21), which has now been added to the web resource data, was calculated as: adjusted *P* = *p* (*m*/*i*), where *p* is the statistical significance test *P*-value, *m* is the number of SmLINC/PC gene pair-wise expression correlation tests under analysis and *i* is the rank of each test, ordered by *P*-value, with 1 the rank of the smallest *P*-value. For each of the three different subsets of correlation analyses from our previous work (7) a different number of tests (*m*) were under analysis; for the analysis of Figure 7A and Supplementary Figure S3 the value of *m* = 4,098,472; for the analysis of Figure 6A the value of *m* = 31,932,880; for the analysis of Supplementary Figure S5 the value of *m* = 37,275,980.

The programing codes for analyzing lncRNAs and deploying the web server for the catalog disclosed herein are available at https://github.com/verjo-lab/Smansoni.lncRNAs and https://github.com/verjo-lab/site_xto, respectively.

Each individual expression profile chart, from the genes present in the co-expression networks built previously (7), was automatically generated through an *ad hoc* R script (https://github.com/verjo-lab/Smansoni.lncRNAs/tree/master/lncRNA-pipeTools) using ggplot2 library (22).

The deployment of co-expression networks onto our web resource was performed through the use of Cytoscape.js JavaScript library (23) (https://github.com/cytoscape/cyjs-sample/wiki).

## Data records

All sequencing data generated by our group can be obtained from the Sequence Read Archive (SRA—NCBI) under the BioProject accession number PRJNA361136. Additional public RNA-Seq data generated by others and used in our *in silico* assays are described in our previous work (7).

A complete list encompassing all significantly correlated (*P*-value <0.05, *r *>* *0.5 or *r *< −0.5) pairs of genes (PC–PC, PC–SmLINC and SmLINC–SmLINC) across five developmental stages (cercariae, somula 3 h, somula 24 h, male and female) was deposited as a compressed tab-delimited file in figshare (https://doi.org/10.6084/m9.figshare.5797257).

The co-expression networks (two Cytoscape .cys files) as well as the thousands of expression profile charts for all SmLINC genes and their correlated mates within the networks were deposited in figshare (https://doi.org/10.6084/m9.figshare.5797290). Furthermore, a web resource was created for easily allowing users to browse and search for their SmLINC genes of interest and visualize their transcriptional profile along with the positively or negatively correlated Smp PC gene mates during parasite development (http://verjolab.usp.br).

The enriched GO terms networks can be navigated at the web resource or its .cys file can be downloaded to be visualized from a local version of Cytoscape (19). The SmLINCs-PC gene co-expression networks .cys file can also be downloaded from our web resource to be visualized in a Cytoscape local version. The Cytoscape software can be downloaded from http://www.cytoscape.org. The *S. mansoni* V5.2 annotated genome sequence (4) and the SmLINC genes loci (7) can be visualized in the *S. mansoni* UCSC-like genome browser (http://schistosoma.usp.br/), which we developed previously (6, 7).

## Funding

This work was supported in part by a grant from the European Union’s Seventh Framework Programme under grant agreement no. 602080 to S.V.A., E.J.R.V. and A.S.A.P. were supported by fellowships from Fundação de Amparo à Pesquisa do Estado de São Paulo (FAPESP 2014/24560-8 and 2016/10046-6). L.F.dS., V.C.M. and G.M.L. were supported by fellowships from Conselho Nacional de Desenvolvimento Cientifico e Tecnologico (CNPq). S.V.A. was also supported by institutional funds from Fundação Butantan and received an established investigator fellowship award from CNPq, Brasil. The funders had no role in study design, data collection and analysis, decision to publish or preparation of the manuscript.

*Conflict of interest.* None declared.
